# Are We Missing Something in the CT-PNS Report? – an Observational Study on the Rate of Reporting the Presence of Dental Disease and the Probable Etiology of Sinusitis on CT Scans

**DOI:** 10.5334/jbsr.2740

**Published:** 2022-11-15

**Authors:** Siddharth Vijayakumar, Sanchanaa Sree Balakrishnan, Rajeev Pulimi

**Affiliations:** 1 Kettering General Hospital, GB; 2 Sri Ramachandra Institute of Higher Education and Research, IN

**Keywords:** odontogenic sinusitis, reporting rate, dental disease, maxillary sinusitis, paranasal sinuses

## Abstract

**Objectives:**

To (i) identify the prevalence of dental disease, (ii) identify the proportion of sinusitis cases that could be considered odontogenic in origin and, (iii) audit the rate of diagnosis of incidental dental disease and odontogenic sinusitis in radiology reports on CT scans covering the maxillary teeth and sinuses.

**Materials and methods:**

Images and reports of CT studies performed in our institution that covered the paranasal sinuses and maxilla were retrospectively audited for documentation of findings pertaining to maxillary sinusitis and maxillary dental disease. Trauma cases, edentulous and pediatric patients and patients without maxillary sinusitis or dental disease were excluded. The etiologies of maxillary sinusitis was defined as likely odontogenic, indeterminate and rhinogenic sinusitis. Only molar and pre-molar tooth disease were considered as these are most commonly in direct contact with the floor of the maxillary sinus.

**Results:**

One-hundred sixty CT studies were reviewed. The prevalence of dental caries and periapical lucency was 80.6% and 15.0%, respectively. The cause of sinusitis was determined to be likely odontogenic in 30.0%, rhinogenic in 33.1% and of indeterminate origin in 36.9%. The rate of reporting dental findings or raising the suspicion of odontogenic sinusitis was 8.5% (n = 14).

**Conclusions:**

Under-reporting of dental disease and odontogenic sinusitis is common. Early recognition results in higher chances of salvaging the diseased tooth, preventing complications and providing appropriate treatment. An urgent and collective effort by the radiological fraternity is warranted to recognize the significance of reporting of dental pathologies, even in CT scans done for other indications.

## Introduction

The importance of identifying dental disease on radiological imaging cannot be overstated.

Firstly, early recognition of incidental dental disease could alert patients to seek dental care in the initial stages of disease, when the treatment would be simple, cost-effective, and associated with higher chances of salvaging the diseased tooth. This potential benefit of early incidental detection on computed tomography (CT) is quite considerable.

Secondly, chronic rhinogenic sinusitis is a common pathology, usually treated with a course of antibiotics; resistant cases may undergo functional endoscopic sinus surgery. However, sinusitis involving the maxillary and other anterior group sinuses may be the consequence of a dental infection. This entity, termed odontogenic sinusitis (OS), refers to reactive inflammatory mucosal thickening in the maxillary sinus caused by the spread of infection from a maxillary tooth due to a breach in the integrity of the intervening bone and the mucoperiosteum. Although a well-established entity, it does not appear to be well known if we consider how under-reported this condition is by radiologists. The pathophysiology and the microbial flora involved in odontogenic and non-odontogenic (rhinogenic) maxillary sinusitis are different, resulting in different management protocols. Treatment of the dental source is an indispensable initial step in treating OS and failure to recognize and eliminate a dental source may lead to failure of any surgery done to treat the sinusitis. Furthermore, many patients with OS do not always present with dental pain and dental findings may be missed on clinical dental evaluation. In contrast, radiological evaluation with CT has very high sensitivity for identifying dental disease, although less than a dedicated dental scan [1]. This certainly appears to place the onus of identifying early dental disease and raising the suspicion of odontogenic sinusitis, at least in part, on the radiologist.

Lastly, grave complications such as orbital cellulitis, blindness and cavernous sinus thrombosis may arise from the spread of dental infections, making their early identification and treatment worthwhile. Additionally, dental disease is known to be implicated in the pathophysiology of cardiac disease and cancer, adding to the potential benefits of its early identification.

Chronic under-reporting of dental disease in radiology reports and the resulting failure in raising suspicion of odontogenic cause of maxillary sinusitis, has led dental surgeons and otorhinolaryngologists to advise their colleagues to read radiological images rather than trust the radiologist’s report – a sad day for the radiological community indeed!

In view of the aforementioned lacunae in the identification of dental disease and consequent sinusitis, our objectives were to identify in our study population (i) the prevalence of dental disease, (ii) the proportion of sinusitis cases that could be considered odontogenic in origin and finally, (iii) audit the rate of diagnosis of incidental dental disease and appropriate recommendations in reports issued by the department of radiology at our institution, on CT scans that covered the maxillary teeth and paranasal sinuses.

## Materials and Methods

We conducted a retrospective study amongst all adult patients (Age > 18y) who had undergone CT studies that included the paranasal sinuses and maxilla, in our institution in the months of October and November 2019. CT studies of patients less than 18 years of age were excluded since their dentition was unlikely to have matured completely.

The total number of CT studies covering the paranasal sinuses and maxilla in adults over the age of 18 years was 340. Out of these, 119 were trauma cases and were excluded. Of the remaining 221 studies, edentulous patients, and those with neither mucosal thickening in maxillary sinus nor dental disease in the maxilla, were excluded. The remaining 160 patients, who had mucosal thickening and/or dental disease, were included.

The age of the patients was between 18–82 years (Mean = 46.4y). The sample included 98 males and 62 females.

CT-paranasal sinuses (PNS) studies and CT-neck and cerebral angiogram (NCA) studies were included. Since the CT brain scan protocol did not always cover the maxillary dentition completely, these were excluded. Helical CT scans were done using GE VCT (64 slice) and Phillips Brilliance (16 slice) scanners, with 0.625 mm and 0.8 mm slice thickness for the 64- and 16-slice scanners respectively, and exposure factors set at 120 kV and 250 mAs. For CT-NCA, the protocol included the anatomy from the level of the vertex of skull to the sternal notch, while for CT-PNS studies the cranio-caudal extent covered the sinuses and the maxillae.

We reviewed the source and reformatted images of the CT scans stored on the institutional PACS to identify mucosal disease involving the sinuses and dental disease involving the maxillary teeth. We then reviewed reports for these scans to audit the reports for identification of dental findings and correlation with maxillary sinus findings.

**Figure 1:**
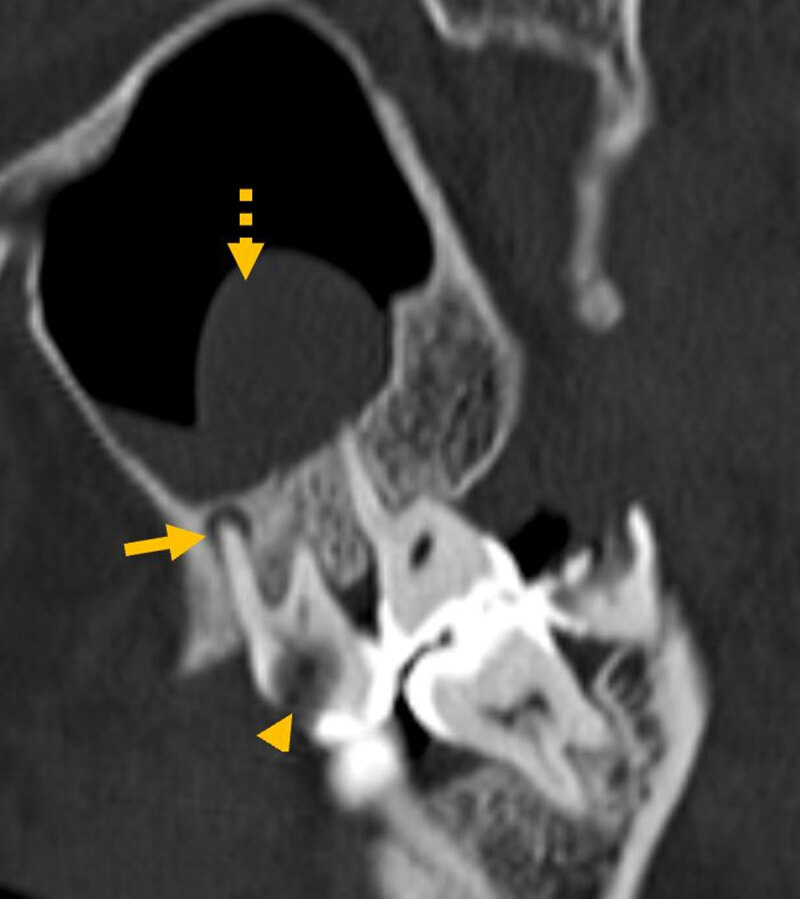
Dental caries (arrowhead) and periapical lucency (arrow) in the same tooth with adjacent polypoidal mucosal thickening (dashed arrow).

We included findings of dental caries, periapical lucency (Figure 1), and projection of the root of a tooth into maxillary sinus.

**Figure 2:**
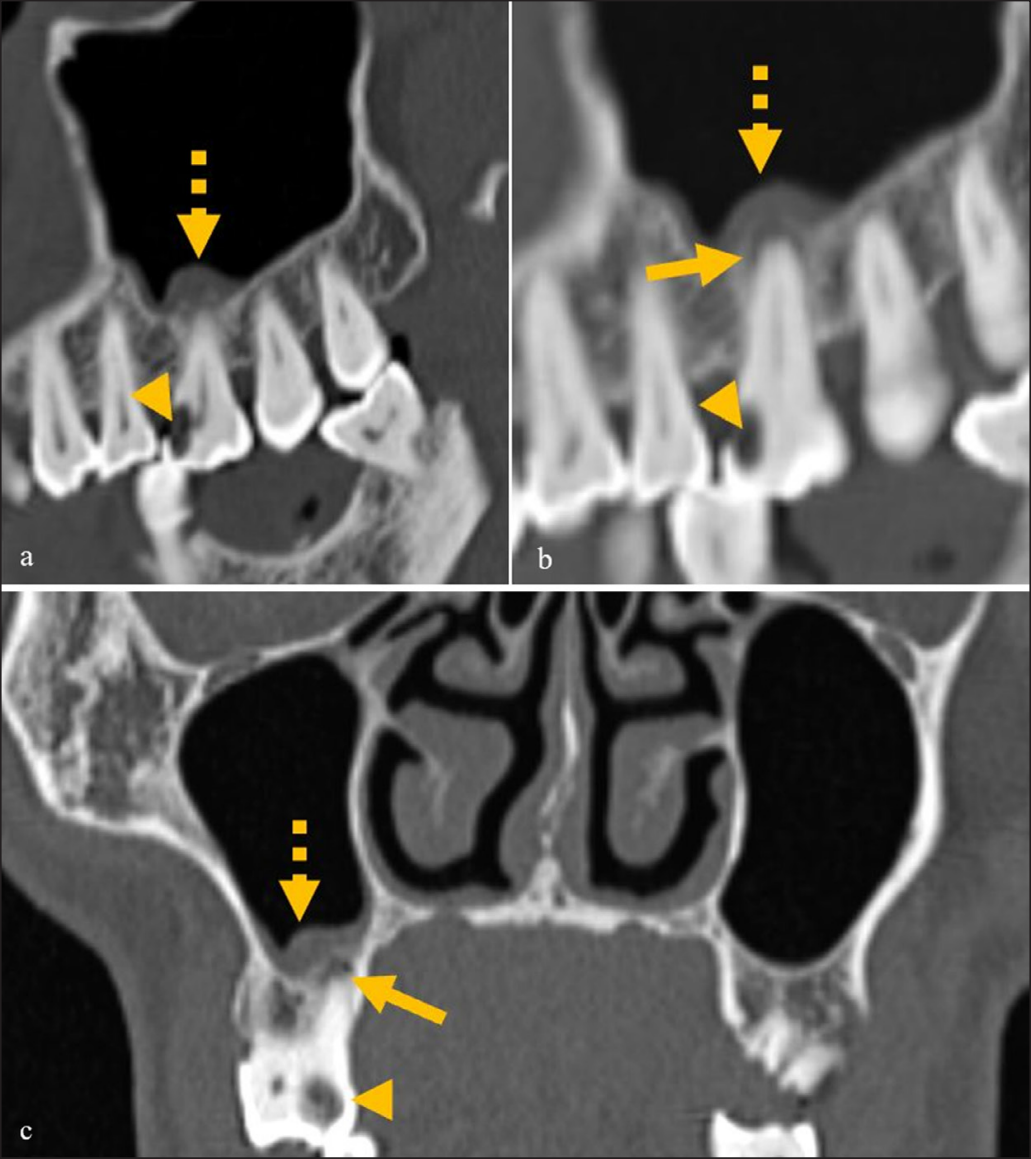
**(a–c)** A case of mild odontogenic sinusitis, showing dental caries (arrowhead) and mild periapical lucency (arrow) in the same tooth with mild mucosal thickening only adjacent to the diseased tooth (dashed arrow).

**Figure 3:**
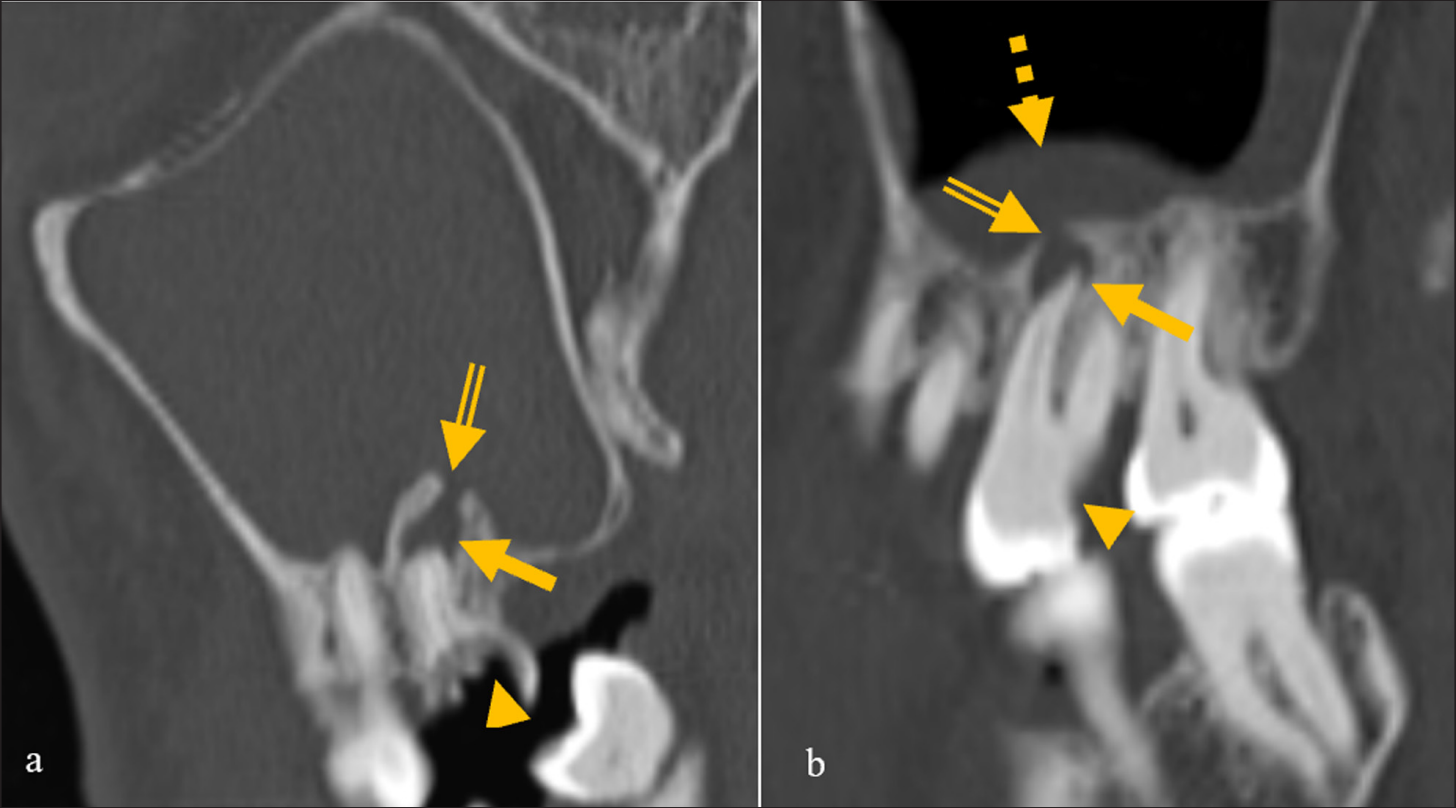
Images of two different patients showing dehiscence of the floor of the maxillary sinus (double lined arrow) and mucosal thickening and the causative diseased tooth with caries (arrowhead) and periapical lucency (arrow).

CT cannot definitively diagnose odontogenic sinusitis. This requires culture of oral microbial flora from a sinus swab. Therefore, we defined the etiologies of maxillary sinusitis as follows:

Likely odontogenic sinusitis: Polypoidal mucosal thickening involving the floor of the maxillary sinus only immediately adjacent to the diseased tooth or presence of an obvious erosion in the floor of the maxillary sinus adjacent to a diseased tooth (Figures 2 and 3).

Indeterminate etiology of sinusitis: Polypoidal mucosal thickening involving the floor of the maxillary sinus, but not limited to the area of diseased tooth.

Rhinogenic (non-odontogenic) sinusitis: No evidence of dental disease in the region of mucosal thickening; mucosal thickening involving all the walls of the maxillary sinus, in a non-polypoidal (uniformly flatted/peripheral) pattern.

We considered only the molar and pre-molar teeth in determining likelihood of odontogenic sinusitis as these were most commonly in direct contact with the floor of the maxillary sinus.

## Observations and Results

### Prevalence of various dental findings (caries, periapical lucency, and projection of tooth into sinus), and distribution of pathology in various tooth types

It was found that 80.6% of patients (n = 129) had one or more carious teeth. The first molar tooth was most frequently diseased (n = 80 patients), followed by the second molar (n = 51), the second premolar (n = 43), the third molar (n = 33), and the first premolar (n = 30). The canine teeth were less commonly involved (n = 21).

In 15.0% of patients (n = 24) periapical lucency was found and was mostly seen around the second molar, followed by the first and third molars respectively, and less commonly, in the premolars (1st > 2nd). Seventy-five percent of these patients had maxillary sinusitis (18/24). Of these, 44.4% (8/18) were found to have sinusitis of likely odontogenic origin, while in 50% (n = 9), origin of sinusitis was indeterminate. In one patient (5.6%) there was rhinogenic sinusitis.

The roots of one or more teeth were projecting into the floor of the maxillary sinus in 45% of patients (n = 72).

### Prevalence, distribution, and etiology of sinusitis

Many authors have defined different criteria for determining maxillary sinus pathology. Using our criteria, as defined in the methodology section, we determined the cause of sinusitis to be likely odontogenic in 30.0% (39/130) of cases, rhinogenic in 33.1% (43/130), and of indeterminate origin in 36.9% (48/130).

Of the 21 patients with unilateral solitary maxillary sinus involvement, 28.6% (n = 6) were likely odontogenic, 42.8% (n = 9) were rhinogenic, and 28.6% (n = 6) were of indeterminate cause. In the nine cases with unilateral multiple sinus involvement, 11.1% (n = 1) were likely of odontogenic origin, while 55.6 % (n = 5) were of rhinogenic origin, and 33.3% (n = 3) were indeterminate. Of the 20 patients with bilateral involvement of only the maxillary sinuses, 45% (n = 9) were of likely odontogenic origin, while 25% (n = 5) were of rhinogenic origin and 30% (n = 6) were indeterminate. In the 80 cases with bilateral multiple sinus involvement, 28.7% (n = 23) were likely of odontogenic origin, 30.0% (n = 24) were of rhinogenic origin, and 41.3% (n = 33) were indeterminate.

In our study population, 13 patients had some form of dental procedure, with treatment material/implants seen on imaging. Of these patients, 30.7% (4/13) patients did not have any sinusitis, while 69.3% (9/13) of patients had maxillary sinusitis. Of these patients 55.6% (5/9) were considered to have odontogenic origin of sinusitis ipsilateral to the prosthesis. Sinusitis was indeterminate for odontogenic origin in 33.3% (3/9) patients and rhinogenic in 11.1% (1/9) patients.

### Rate of reporting

The overall rate of reporting dental findings or raising the suspicion of odontogenic sinusitis was found to be 8.5% (n = 14). In 91.5% of cases, dental findings including caries, periapical lucency, or probable association of dental findings to mucosal thickening in the floor of the maxillary sinus, was not commented upon.

## Discussion

The incidence of dental disease in India is high. The Global burden of disease survey (2016) showed that 31% males and 33% females in India had carious permanent teeth [2]. In our study, we found that the proportion of patients with caries was much higher (80%). This ranged from early-stage disease with just enamel loss to advanced stage caries with extensive crown destruction.

The existing literature showed significant variation in the range of prevalence of periapical pathology. Maillet et al. found that the prevalence of periapical lucency in their study was 65.4%, whereas Bajoria et al. reported a prevalence of 36.1% [3,4].

Proximity of the apex of tooth to the floor of the maxillary sinus is an important factor that weighs in the spread of infection. Normally, the cortex of the maxilla acts as effective barrier to the spread of infection from the tooth to the maxillary sinus. However, if the apex of the tooth is close to, abutting, or projecting into floor of the maxillary sinus, only the thin Schnederian membrane may be left separating the apex of the tooth and the sinus cavity, predisposing to the spread of infection. The floor of the maxillary sinus is closest to the roots of the maxillary molar and premolar teeth with a mean distance of 1.97 mm [5]. This proximity explains why infections of the molars and premolars can easily spread to the maxillary sinus. In our study we found that in 45% of patients the tooth was abutting or projecting through the floor of the maxillary sinus. Thus, identifying and reporting dental pathology is essential to diagnose or rule out OS. Whyte et al. stated that mucosal thickening was ten times more common in individuals with periapical lesions, demonstrating the far-reaching effects of dental infection and the need to address it [6].

Although, Lindahl et al. reported that a relation to dental infections was found in 47% of cases of chronic maxillary sinusitis as early as in 1982, the incidence of OS has long been underestimated at 10–12% [7,8]. Much of recent literature, however, estimates the prevalence more accurately. The prevalence of OS was found to be 51.8% by Maillet at al. (2011), 31% by Nascimento et al. (2016), 40% by Fredriksson et al. (2017), and 48% by Ly (2018) [3,9,10,11].

In corollary, studies have also shown that 70–80% of teeth with periapical lesions are associated with OS [12,13]. In our study 75% of cases with periapical pathology had maxillary sinus changes, of which 44.4% were determined to be of likely odontogenic origin and 50% were indeterminate, while only a meagre proportion was rhinogenic. In their 2013 article, Chapman et al. described unilateral focal related to periapical pathology as highly suspicious for a casual relation [8]. Although unilateral maxillary sinusitis in spatial relation to a diseased tooth is pathognomonic for odontogenic etiology, bilateral maxillary sinus involvement as well as multiple sinus involvement can also occur [6,14,15].

Apart from naturally developing dental disease, multiple studies have found iatrogenic etiology to be a significant contributor to odontogenic sinusitis [16,17,18]. In our study, in patients with a dental prosthesis or implant in the maxillae, we determined the cause of sinusitis was likely odontogenic in 55.6% in those with dental prosthesis, while 33.3% were indeterminate. Only one appeared to be clearly rhinogenic. This agrees with existing literature. Hence, special attention must be given to look for endo-antral syndrome in patients with odontogenic sinusitis who have had dental procedures and implants in the past.

The overall rate of reporting dental findings and consideration of odontogenic sinusitis in our study was a dismal 8.5% (n = 14). In 91.5% of cases, dental findings including caries or periapical lucency or probable association of dental findings to mucosal thickening in the floor of the maxillary sinus were not commented upon.

It stands to reason that early recognition of incidental dental disease could potentially allow patients to seek dental care in the early stages of disease – at which point the treatment protocol would be simpler and less expensive, apart from the higher likelihood of the diseased tooth being salvaged. While a CT of the head and neck regions would not be advocated merely for the detection of early-stage dental disease, there is potential for improvement in quality of life if a patient should choose to seek early help for incidentally detected disease.

Odontogenic sinusitis differs from rhinogenic sinusitis in several respects. Sinusitis due to odontogenic cause may be more severe due to the formation of biofilms [12]. Also, the microbes involved in odontogenic sinusitis are different from those involved in rhinogenic sinusitis [19]. Streptococcus pneumoniae, Hemophilus influenzae, Moraxella catarrhalis, Hemolytic streptococci, Microaerophilic streptococci, and Staphylococcus aureus are implicated in rhinogenic maxillary sinusitis. In contrast, anaerobic gram-negative oral flora including Peptostreptococcus, Fusobacterium, pigmented Prevotella, and Porphyromonas spp, predominate in dental infections and OS. Thus, OS harbours different microbes that demands vastly different antibiotics.

The overall approach to the management of OS and non-OS is also different. Treatment of the dental source is an indispensable initial step in treating odontogenic sinusitis [20]. Several studies state that failure to recognize and eliminate a dental source first may lead to failure of functional endoscopic sinus surgery (FESS) performed to treat the sinusitis [21]. The American Association of Endodontists recommends the treatment of primary endodontic infection before undertaking FESS. Newsome found that 15–20% of OMS may resolve with an antibiotic regimen, ruling out the need for surgery in some cases [14]. In their study, Tomomatsu et al found that 51% of patients improved with dental treatment and antibiotics [22]. In another study by Safidi et al., OS involving the frontal sinus was found to resolve without frontal sinusotomy once the dental infection was treated and middle meatal antrostomy was performed [23]. They stated that there was no justification for performing frontal sinusotomy for OS involving the frontal sinus and went on to state that it was in fact contraindicated.

The prerequisite to recognizing possible OS is, understandably, identifying an odontogenic source, which implies assessing the maxillary teeth for dental disease. Hence, the two entities are considered in tandem.

Another worrisome trend is the growing body of otolaryngological and dental literature, as described by Whyte et al. in 2019, that note that radiology reports tend to overlook dental pathology. Wang et al. noted that only 65% of radiology reports mentioned OS [6]. Another otolaryngological article by Newsome in 2019, found that radiologists missed OS 60% of the time [14]. These papers go on to suggest that dental surgeons and ENT surgeons ought to review the radiology images themselves. Whyte et al. noted that most literature on OS was from specialties other than radiology, reflecting the decreased attention the entity has received from imaging specialists over the decades [6]. A study by Hammoud in 2018 focused exclusively on assessing the rate of reporting of dental disease and evaluating whether the addition of a passive field in the radiology report format would encourage radiologists to report dental disease [24]. They found that only 11% of initial reports mentioned dental disease and that addition of a field for dental disease in the report template did not make a significant difference in reporting rate – a tendency that perhaps requires voluntary effort and a change in the mindset of radiologists.

Also, OS may not present with dental pain. It has been found that less than one third of patients with odontogenic sinusitis present with dental pain [6,21]. Matsumoto in 2015 found that in 86% of cases, clinical dental check failed to identify early dental disease [25]. In contrast, radiological evaluation with CT is the gold standard and has 100% sensitivity for identifying dental disease [1,26]. Thus, radiology as a specialty should be playing a key role in identifying these patients.

Lastly, many serious conditions such as orbital cellulitis and blindness, subdural empyema and cavernous sinus thrombosis might complicate OS [27,28,29]. Additionally, dental disease is known to be associated with more core health conditions including cardiac disease. Additionally, early identification of incidental dental disease could potentially help reduce the incidence of these conditions, making the identification and treatment of the dental infections on CT a significant step towards holistic healthcare [12,30].

The main limitation of this study is that it was conducted only in one institution and reviewed the reports of the radiologists there alone. Although studies have been conducted in the past, studies from multiple institutions would throw more light on the pervasive nature of the issue. Also, we included CT-PNS studies, which have slightly lesser sensitivity than the dedicated dental CT scan, albeit using a lower radiation dose.

## Conclusion

CT is undoubtedly the ideal modality to identify dental disease. Although the incidence is high and clinical implications profound, there is a persistent and pervasive neglect in the reporting of incidental dental pathologies and odontogenic sinusitis on CT studies. This has led dental surgeons and otorhinolaryngologists to believe, and advise in their literature, that learning to view the radiological images themselves is a better alternative than trusting the radiologist’s report in this regard. An urgent and collective effort on the part of the radiological fraternity is warranted, to recognize the significance of mandatory reporting of dental pathologies. Their impact on a patient’s quality of life is evident as it facilitates identification and management of disease in the early stages – before symptoms or complications develop – which is understandably simpler and more cost-effective. Further, reporting of odontogenic cause of maxillary sinusitis has ramifications on the clinical management of patients who present with symptoms of sinusitis, since there is a stark difference in treatment of odontogenic and rhinogenic sinusitis.
